# Middle-term bowel function and quality of life in low-type anorectal malformation

**DOI:** 10.1186/s13052-019-0701-3

**Published:** 2019-08-13

**Authors:** Haiqing Zheng, Guangjian Liu, Zijian Liang, Yunpei Chen, Zhe Wen, Jiakang Yu, Xiaogang Xu, Huiying Liang, Yong Wang

**Affiliations:** 1 Institute of Pediatrics, Guangzhou Women and Children’s Medical Center, Guangzhou Medical University, Guangzhou, Guangdong China; 2 Department of Pediatric Surgery, Guangzhou Women and Children’s Medical Center, Guangzhou Medical University, 9 Jinsui Road, Guangzhou, 510623 China

**Keywords:** Low-type anorectal malformations, Bowel function, Quality of life, Vestibular fistula, Perineal fistula

## Abstract

**Background:**

Low-type anorectal malformations (ARMs) are considered benign; however, in China, data regarding such conditions are limited. Thus, this study aimed to assess the middle-term bowel functions and quality of life (QOL) among children with low-type ARM.

**Methods:**

Children > 3 years of age who underwent treatment for low-type ARM (vestibular fistula [VF] and perineal fistula [PF]) during 2013 and healthy children were included. The children were interviewed during their outpatient visits. The primary outcome measures were bowel function, as assessed using the Baylor Continence Scale (BCS), and QOL, as measured using the Pediatric Quality of Life Inventory (PedsQL 4.0).

**Results:**

A total of 82 patients responded; mean patient age was 6.8 (range, 3–12) years. Mean BCS score in the control group (7.94 ± 4.74) was significantly lower than that in the VF (18.69 ± 11.11, *P* < 0.001) and PF (15.47 ± 6.50, P < 0.001) groups. However, there were no differences in PedsQL 4.0 scores among the groups. The patients scored the lowest for emotional function and the highest for physical function. Nearly all measurements of QOL significantly decreased with increased BCS score.

**Conclusions:**

Children with low-type ARM can achieve good bowel control and QOL. However, although ARMs are benign, several children with this condition suffer from anal function problems that affect QOL. Redo operations, mislocated anus, and incorrect constipation treatment are the iatrogenic causes of fecal incontinence.

## Background

Most pediatric surgeons use the term “low-type” anorectal malformations (ARMs) in cases of rectoperineal or rectovestibular fistula. Low-type ARMs comprise an important subset of the distinct types of anomalies in both sexes, accounting for approximately half of all ARMs [1]. The surgical correction of low-type ARMs is usually performed early in life, with surgical therapy offering almost universal survival.

Traditionally, the long-term results of low-type ARMs have been favorable in most patients [2–4]. Thus, this study aimed to investigate whether low-type ARMs are associated with favorable outcomes, particularly in terms of middle-term bowel function and quality of life (QOL) among surgically treated children as well as the impact of bowel function on QOL. We hope that the information obtained from this study will be valuable for pediatric surgeons in evaluating long-term recovery from low-type ARMs.

## Methods

### Sample

One-hundred children who underwent surgery for ARM in 2013 at the Guangzhou Women and Children’s Medical Center (GWCMC) were invited to participate in the study. The children were diagnosed via clinical examination. Children younger than 3 years and those with hypothyroidism, hypokalemia, hypomagnesemia, hypophosphatemia, hypercalcemia, spina bifida, myelomeningocele, and tethered cord were excluded. The control group comprised 86 healthy children without bowel dysfunction (52 boys and 34 girls), with a median age of 6.0 (range, 3–12) years, from a primary school during the same period.

### Surgical management

**Fig. 1:**
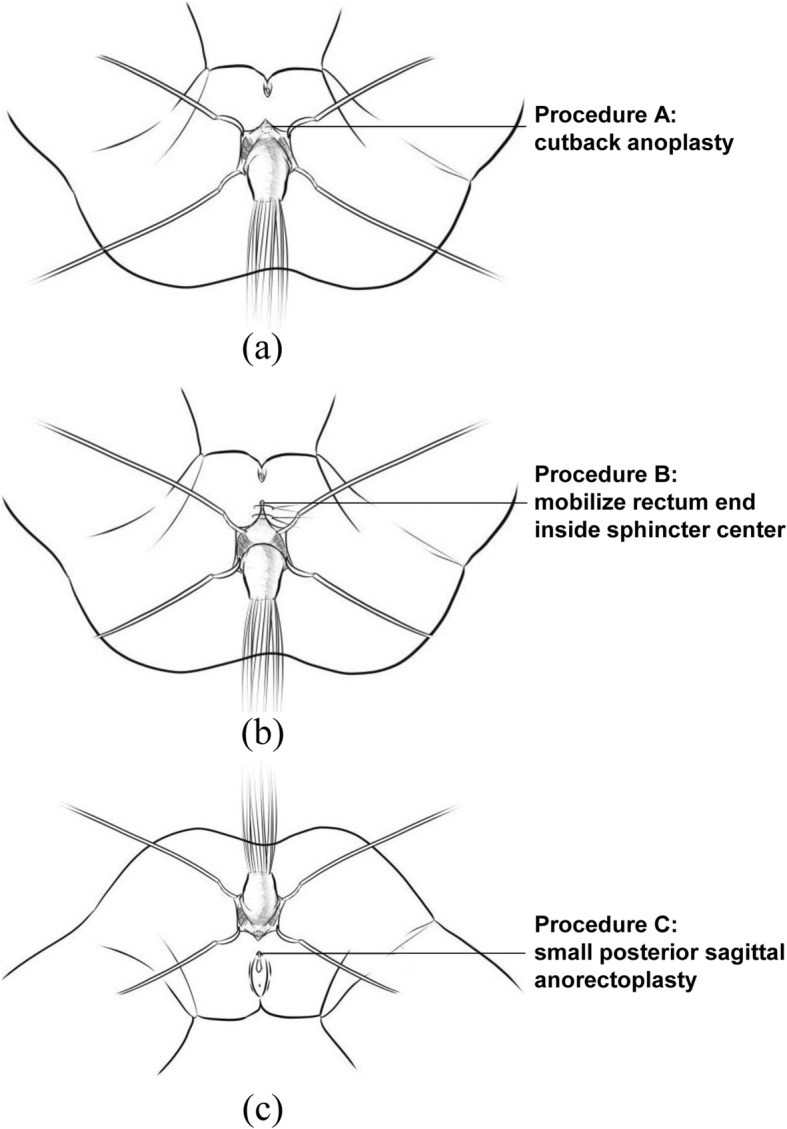
Procedures performed for (a) perineal fistula (PF) located inside the sphincter, (b) PF located partly inside the sphincter, and (c) vestibular fistula (VF)

For ARM with a rectoperineal fistula, we preferred cutback anoplasty if the rectum was located inside the voluntary sphincter funnel (Procedure A in Fig. 1a). If the rectum was located only partly inside the sphincter, we mobilized the whole terminal end of the rectum and placed it inside the center of the sphincter while the patient was in lithotomy position (Procedure B in Fig. 1b). For ARM with a vestibular fistula (VF), we performed small posterior sagittal anorectoplasty (PSARP) with partial incision in the perineal body (Procedure C in Fig. 1c). Our approach aimed to use minimal surgical interventions while preventing unnecessary invasive surgery.

### Procedures

The research ethics board of GWCMC approved this study. The children and their parents were approached at the time of their clinical appointment by an independent researcher and were recruited in the study after providing informed consent. The participants were invited for an outpatient visit and were examined cross-sectionally during their outpatient visit between 2017 and 2018. The patients were interviewed by trained nurses and were asked to complete the Baylor Continence Scale (BCS) and the Pediatric Quality of Life Inventory (PedsQL 4.0). We compared the bowel function and QOL between patients with VF and perineal fistula (PF) ARMs. Data about age, sex, associated anomalies, and surgical methods were collected from the medical records. The primary outcome measures were bowel function score and QOL that were assessed using BCS and PedsQL 4.0, respectively.

### Measurements

The study package contained two questionnaires: the BCS for bowel function and PedsQL 4.0 for QOL [5, 6]. The BCS includes 23 questions that have been designed based on clinical experience. The BCS scores range from 2 to 84, with lower scores reflecting better social continence. The questionnaires can be completed by children or their parents without clinical examination and is observer-independent. The PedsQL 4.0 is a modular instrument designed to measure QOL in healthy and acutely or chronically ill children and adolescents aged 0–18 years [7]. The PedsQL 4.0 inventory was used to test health-related QOL in children using multidimensional parent proxy reporting (for children aged 4–7 years) and child self-reporting (for children aged 8–17 years) with respect to the 23 parameters. It assesses both physical health (eight items) and psychosocial health, which include emotional, social, and school performance (five items each). For this analysis, total parent-reported QOL scores as well as physical (physical function) and psychosocial (emotional, social, and school functioning) QOL scores were examined.

### Statistical analysis

Data were analyzed using the Statistical Package for the Social Sciences software version 19.0 (IBM SPSS for Windows; IBM Corporation, Somers, NY, the USA) and GraphPad Prism version 5.0 (La Jolla, CA, the USA). A two-sided *P* < 0.05 was considered statistically significant. Categorical variables were presented as n (%) and were compared using the chi-square test. Continuous variables were presented as mean and standard deviation or median (interquartile range) and were compared using *t*-test or analysis of variance. The correlation between the characteristics of the patients as well as bowel function and QOL were assessed using the Pearson’s correlation coefficient. Linear regression analysis was carried out to assess the association between BCS score and QOL (measured using PedsQL 4.0 and each dimension score). The models were adjusted for sex, current age, age during surgery, weight during surgery, body mass index (BMI), surgical procedure, and presence or absence of comorbidities, and 95% confidence intervals (95% CIs) were calculated.

## Results

### Patient characteristics

Of the 100 patients, four presented with hypokalemia and one with spina bifida. A total of 13 patients were lost to follow-up, and the remaining 82 patients were included in the case group. Table 1 shows the demographic data of the patients. A total of 168 participants (39 with VF, 43 with PF, and 86 healthy children) were finally included in the study; most patients were girls (55.9%). Among the three groups, no significant difference was observed in terms of age (*P* = 0.356) and BMI (*P* = 0.667). Children with VF were older during surgery (*P* = 0.001); thus, they had higher body weights during the time of surgery (*P* = 0.013) than those with PF. All patients with VF underwent reconstruction via PSARP, whereas only 6 (14.3%) patients with PF underwent reconstruction via PSARP. Moreover, 15 (35.7%) patients underwent reconstruction via B and 21 (50.0%) via C (*P* < 0.001). The proportions of associated anomalies were not significant between the PF and VF groups (*P* = 0.425). Two patients underwent anal reconstruction and the pull-through procedure for secondary megacolon.

**Table 1:** Characteristics of the study population

Variable	Vestibular fistula (*n* = 39)	Perineal fistula (*n* = 43)	Control group (*n* = 86)	*P*
Gender				
Boy	0(0%)	22(51.2%)	52(60.5%)	< 0.001
Girl	39(100%)	21(48.8%)	34(39.5%)	
Current Age (year)	7.17 ± 2.06	6.53 ± 1.68	6.68 ± 2.28	0.356
BMI (Kg/m^2^)^b^	15.73 ± 4.04	16.34 ± 2.81	15.73 ± 3.21	0.667
Weight at surgery(Kg)	7.25 ± 5.27	5.09 ± 3.30	–	0.013
Age at surgery (year)	1.11 ± 2.30	0.51 ± 1.78	–	0.001
Surgical procedures^c^				
Procedure A	0(0%)	21(50.0%)	–	
Procedure B	0(0%)	15(35.7%)	–	
Procedure C	39(100.0%)	6(14.3%)	–	< 0.001
Comorbidity				
Circulatory system	2 (5.1%)	7(16.3%)	–	0.425
Urinary system	1 (2.6%)	1 (2.3%)	–	
Others	3 (7.7%)	2 (4.7%)	–	
No	33 (84.6%)	33 (76.7%)	–	

^a^ Data were presented as n (%) and mean ± standard deviation

^b^ BMI was calculated using current weight and height

^c^ Procedures performed for perineal fistula located inside the sphincter (Procedure A), perineal fistula located partly inside the sphincter (Procedure B), and vestibular fistula (Procedure C). See Methods for details

### Bowel function

**Fig. 2:**
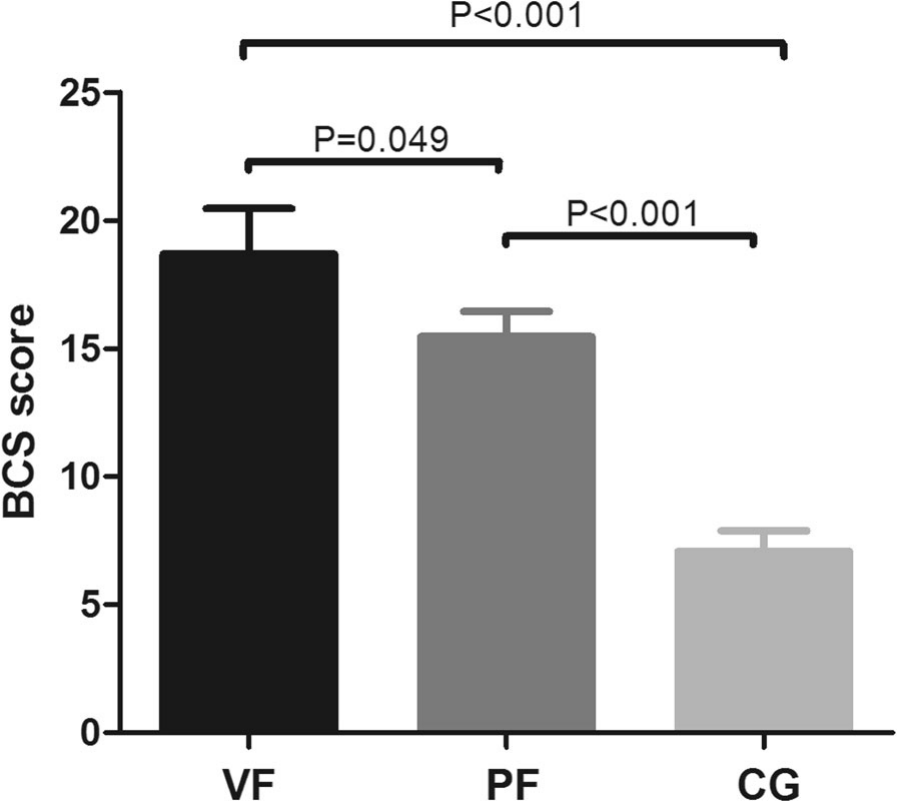
Comparison of BCS score among children with VF and PF and healthy children. The column height represents mean value; the error bar represents standard deviation. *P* value was obtained using the Kruskal–Wallis test. VF, Vestibular fistula; PF, perineal fistula; CG, control group; BCS, Baylor Continence Scale

**Fig. 3:**
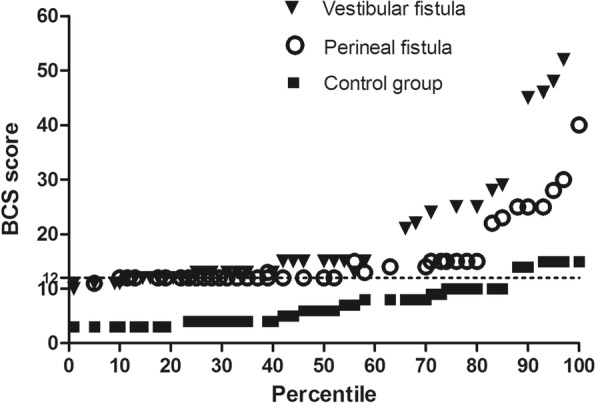
Percentile distribution of bowel function scores among children with VF and PF and controls. VF, vestibular fistula; PF, perineal fistula

As shown in Fig. 2, the mean BCS score of the control group (7.94 ± 4.74) was significantly lower than the groups with VF and PF (18.69 ± 11.11 vs 15.47 ± 6.50). In addition, the scores of children with VF were higher than those with PF (*P* = 0.049). The median scores of children with VF, those with PF, and controls were 13 (interquartile range [IQR], 12.0–22.0), 12 (IQR, 12.0–15.0), and 7.0 (IQR, 4.0–10.0), respectively. The percentile distribution of individual scores has also been presented. Half of the patients (53.6%) had a BCS score > 12, which is the median BCS score in children with PF. In fact, 27 (69.2%) children with VF, 17 (39.5%) children with PF, and 14 (16.3%) healthy children had a BCS score > 12 (*P* < 0.001). Four children in the VF group and one child in the PF group had a BCS score > 40, whereas none of the children in the control group had a BCS score > 40 (Fig. 3). Baylor’s Questions 4, 12, 13, 16, 18, 20, and 21 had a higher score. In patients with higher scores, constipation was the main complaint. Patients with a score between 20 and 30 often complained about pain during defecation and blood in the stool. These children often chose lactulose, glycerol, or even Chinese medicine to help with bowel movements, and only few used enema or irrigation. Two children with VF who have 45 and 52 points and one child with PF who obtained 48 points presented with long-term constipation.

### QOL

**Fig. 4:**
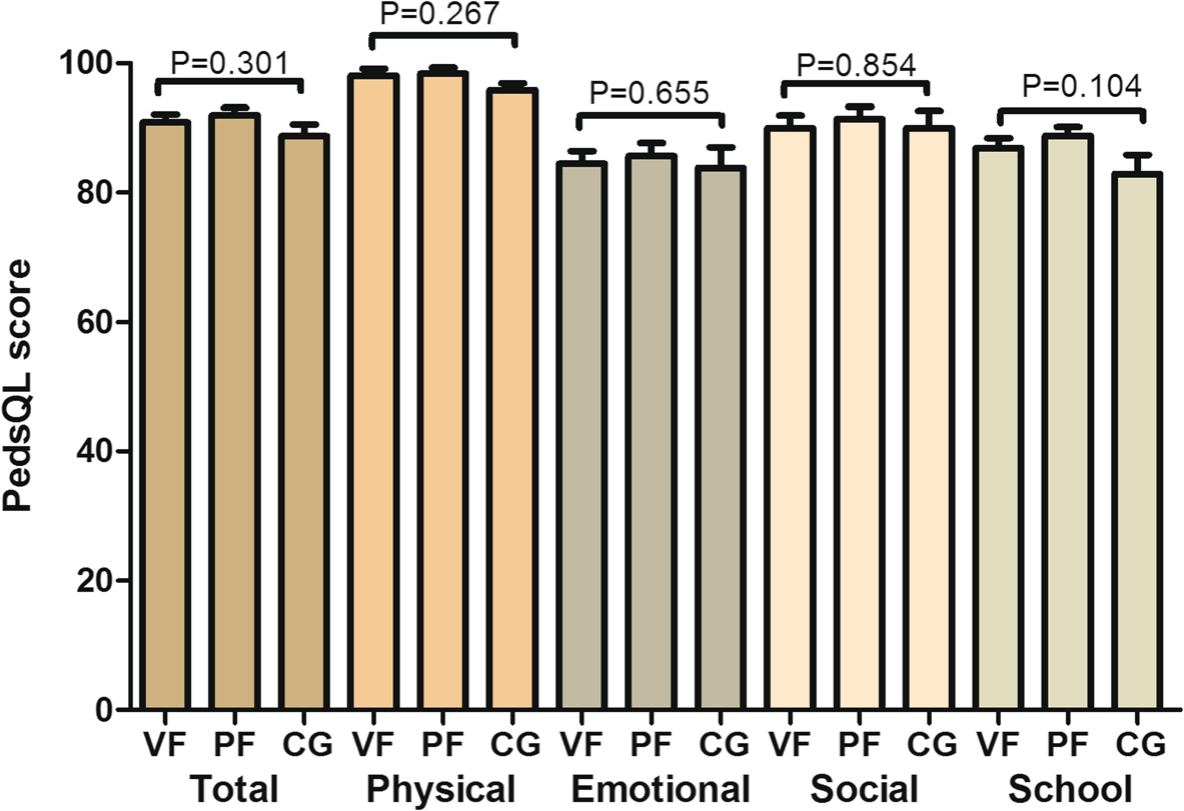
Comparison of the total PedsQL score and scores of each dimension among children with VF and PF and healthy children. The column height represents mean value; the error bar represents standard deviation. *P* values were obtained using the Kruskal–Wallis test. VF, vestibular fistula; PF, perineal fistula; CG, control group; PedsQL, Pediatric Quality of Life Inventory

**Fig. 5:**
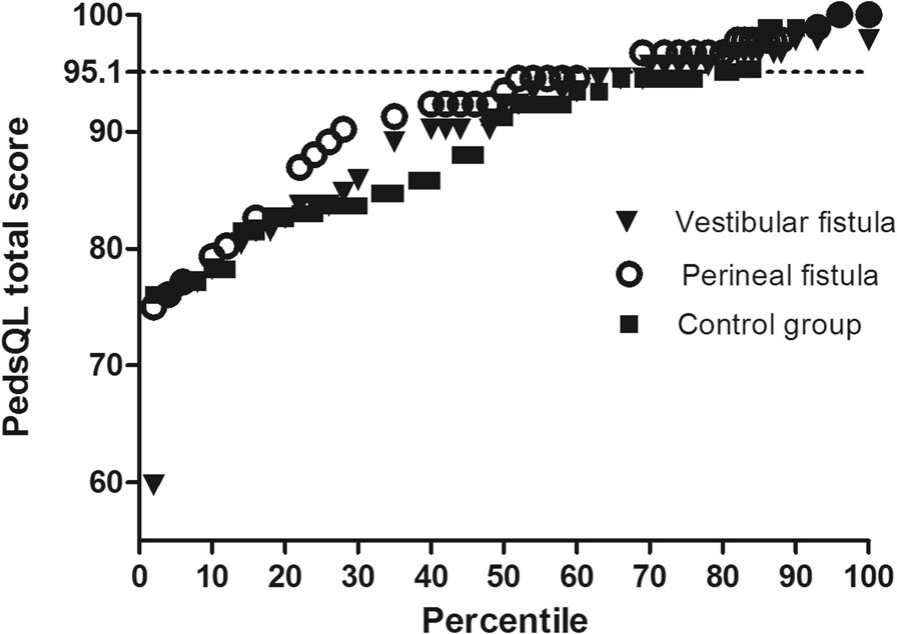
Percentile distribution of quality of life scores among children with VF and PF and controls. VF, vestibular fistula; PF, perineal fistula

As shown in Fig. 4, no difference was observed in PedsQL score among the three groups. The mean total score in healthy children, those with VF, and those with PF were 89.10, 90.89, and 92.03, respectively. The median scores of children with VF, those with PF, and healthy children were 93.47 (IQR, 85.87–96.74), 95.65 (IQR, 88.04–97.83), and 89.02 (IQR, 82.53–95.56), respectively. The distribution of individual total PedsQL 4.0 score is also presented. Overall, the total score was high; only two patients had a score < 70. The total PedsQL 4.0 scores in 21 (53.8%) children with VF and 22 (48.8%) children with PF were < 95.10, which were higher than that of the 48 (69.6%) healthy children (*P* = 0.820) (Fig. 5).

### Relationship between bowel function and QOL

**Fig. 6:**
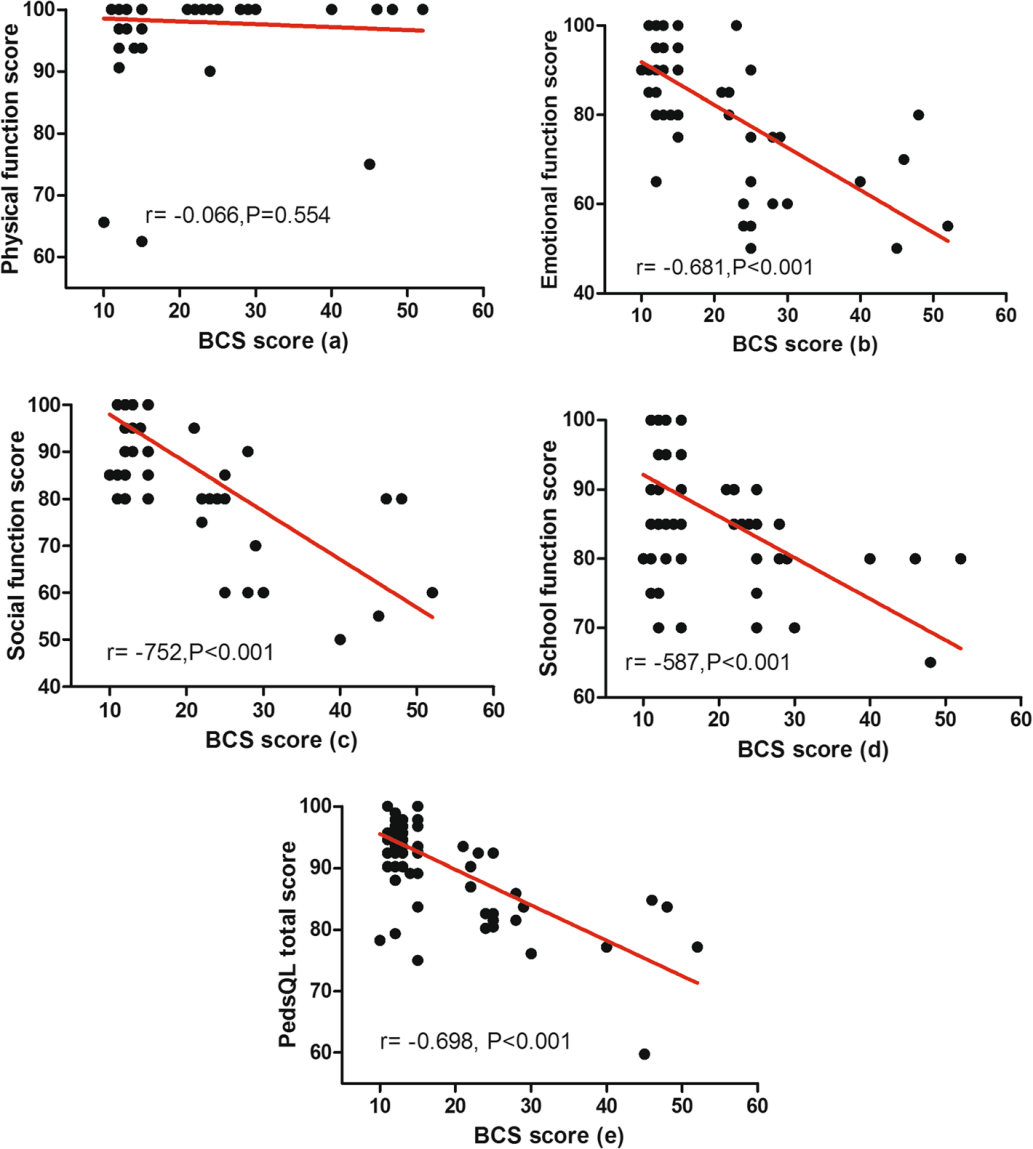
Relationship between BCS score as well as physical (a), emotional (b), social (c), and school function scores (d) and the total PedsQL score (e) in patients with ARM

The correlation analysis showed that BCS score was not correlated to current age, weight during surgery, and age during surgery. In addition, no correlations were found between the characteristics of the patients and total PedsQL score (Table 2). The relationship between anal function and QOL among children with ARM is presented in Fig. 6. The total PedsQL 4.0, emotional, social, and school function scores decreased as the BCS score increased. We used the total PedsQL 4.0 score and the scores of each dimension for dependent variables in the linear regression analysis (Table 3). After adjusting for gender, current age, age during surgery, weight during surgery, BMI, surgical procedure, and comorbidity, nearly all measurements of QOL, including total scale, emotional, social, and school function scores, significantly decreased as the BCS score increased (β, − 0.58 and 95% CI, − 0.72, − 0.44; β, − 0.96 and 95% CI, − 1.19, − 0.72; β, − 1.01 and 95% CI, − 1.31, − 0.89; and β, − 0.59 and 95% CI, − 0.78, − 0.39, respectively). However, the BCS score was not associated with physical function (β, − 0.03 and 95% CI, − 0.20, 0.13).

**Table 2:** Correlations between the characteristics of the patients and BCS score and PedsQL total score

	BCS score	PedsQL total score
Current age (year)	0.04	−0.06
Weight at surgery (Kg)	−0.03	−0.05
Age at surgery (year)	0.02	−0.10

Abbreviations: BCS, Baylor Continence Scale; PedsQL, Pediatric Quality of Life Inventory

**Table 3:** Adjusted estimates (β) for the association between BCS score as well as the total PedsQL score and physical, emotional, social, and school function scores in patients with ARM^a^

PedsQL	β	SE	95%CI	P
Total Score	−0.58	0.07	−0.72, − 0.44	< 0.001
Physical function	− 0.03	0.08	− 0.19, 0.13	0.745
Emotional function	−0.96	0.12	−1.19, −0.72	< 0.001
Social function	−1.01	0.11	− 1.31, −0.89	< 0.001
School function	−0.59	0.09	−0.78, − 0.39	< 0.001

^a^BCS score was considered an independent variable after adjusting for gender, current age, age during surgery, weight during surgery, BMI, surgical procedure, and comorbidity

Abbreviations: CI, confidence interval; BCS, Baylor Continence Scale; PedsQL, Pediatric Quality of Life Inventory

## Discussion

This study aimed to identify the bowel function and middle-term QOL of patients with low-type ARM, which is traditionally considered a benign abnormality [8]. Although the outcomes of low-type ARM are generally favorable [4], some patients still present with poor outcomes. This study provides contemporary data about middle-term functional and QOL outcomes in children with low-type ARM. Moreover, according to a dropout analysis, responders and non-responders had equal age and sex distribution and disease characteristics; thus, a significant selection bias is unlikely to occur.

The validated BCS tool was used to assess bowel function, and it can differentiate between normal children and those with various comorbidities. Lower scores indicate better social continence. Normal children without anatomic or functional continence problems had a mean BCS score of 11.5 [9], whereas all our patients with low-type ARM had a mean score of 17.7. Constipation was the most common early functional problem (40%) in patients with low-type anomalies [10]. When the malformation is lower, the incidence of severe constipation is higher, leading to overflow pseudo-incontinence [11]. Peña has described various degrees of postoperative constipation in 70% of patients with VF and in 50% of patients with low-type ARM [12]. These findings have their roots in the pathogenesis of ARM. First, Holsehneider and other scholars have recently pointed out that disturbances in rectal end innervation caused by surgery might lead to a high incidence of constipation [13]. Second, in 2007, Senel et al. [14] have hypothesized that constipation may be correlated to rectoanal inhibitory reflex. Abnormal colonic motility was also found in patients with ARM. Most patients with ARM present with disturbance in sophisticated bowel motility mechanism [15]. In fact, constipation is the main cause of fecal soiling, and once constipation is treated, continence and defecation pattern return to normal. In our cases, early treatment for postoperative constipation is non-standard. Some patients are treated with glycerine enema, and in some cases, traditional Chinese medicine is prescribed. Few children were treated according to the international constipation treatment protocol. Among our patients, five had a low Baylor score, of which one experienced soiling from long-term constipation. Four patients had normal spinal cord (no tethered cord) that can affect bowel control; thus, we believe that bowel dysfunction is attributed to iatrogenic causes in these patients. Among them, two had anus located outside of the sphincter. The surgeons might have performed the cutback procedure instead of the correct procedure (as shown in Fig. b). The other two patients underwent PSARP and the pull-through procedure for secondary megacolon. We believe redo operations, damage in the sphincter, mislocated anus, and incorrect treatment of constipation are the causes of fecal incontinence. In patients with fecal incontinence, the bowel management program is usually initiated [16]. Patients who require BMP therapy will visit the clinic daily for the whole week. Each patient is provided with enema, and home therapy is then performed daily. In three patients with confirmed fecal incontinence, the Malone procedure was conducted. However, only few parents provided consent for such procedure as most of them believe that their children can have normal bowel control as they get older.

The mean BCS score was higher in patients with VF than in those with PF, indicating that the bowel function of patients with PF was better than that of patients with VF. Intergroup differences can be explained by three facts. First, innervation may be involved; thus, all patients with VF (100%) and > 70% of patients with PF showed varying degrees of innervation abnormalities [17]. Second, in PF cases, the anal canal is usually located at least partially inside the voluntary sphincter funnel and is consequently managed surgically using less invasive approaches than PSARP. Therefore, in VF cases, the bowel terminates completely outside the sphincter. Third, this may be associated with the choice of procedure; that is, we prefer the PSARP procedure for patients with VF as some studies have shown that it is the main cause of constipation in ARM [18, 19]. However, this result remains controversial. In our VF cases, we performed minimal PSARP procedure without a protective colostomy, whereas some surgeons perform anterior sagittal anorectoplasty without colostomy. However, in this study, the number of patients is not sufficient to perform comparison and analysis, and we cannot conclude whether the PSARP procedure will cause constipation in our patients. In our PF cases, the treatment approach has involved individualized, minimally invasive perineal procedures that achieve satisfactory stool passage. The cutback procedure is preferred if the anus is surrounded by the sphincter. However, the key point is that the rectum should be exactly at the center of the anal sphincter.

QOL has been an important endpoint in the medical care of patients with ARM who have psychological, behavioral, or developmental problems and who experience significantly lower QOL than children without such problems. Poor outcomes have been correlated to neurological damage and mental retardation [20] or insufficient long-term follow-up and care of patients [21, 22]. In our study, a clinically relevant impairment in QOL was observed based on child self-reported total scores, particularly emotional and social score. Most likely, at least some duration of bowel dysfunction will make them feel different from their peers, causing difficulty in feeling like a part of the crowd. The fact that impairment in QOL is mostly in the psychosocial domain is not surprising. However, the QOL of the affected group was not significant differently from that of the control group. Two reasons are associated with this phenomenon. First, the participants in the ARM and normal groups were recruited from different areas. Since employment, income, education, and healthcare levels differ among regions and locations, these family differences may impact individual QOL. Second, although patients present with congenital malformations that can cause fecal incontinence, often having irreversible consequences, the participants (or their parents) in this study had positive perceptions of their QOL since their bowel control was significantly better than expected at the beginning. A previous report has shown similar results [23]. In addition, the low-type ARM is often a part of a malformation complex [24, 25]. The associated anomalies can significantly impact the QOL of patients. Cardiac (15–40%) and genitourinary malformations, including vesicoureteral reflux (15–30%), are most commonly observed [25, 26]. In our study, 23.3 and 15.4% of patients with PF and VF, respectively, presented with circulatory and urinary problems or other comorbidities.

Moreover, this study showed that functional scores were significantly correlated to QOL in emotional, social, and school areas. More severe functional situations resulted in a lower QOL. Bad bowel function, particularly fecal incontinence, is highly correlated to QOL. Most adult patients with ARM had no social problems in occupational or student life if they gained good bowel control. Grano has investigated how fecal incontinence may influence the different aspects of QOL in children and adolescents with ARMs [27]. In our study, only few patients presented with poor bowel control but have high QOL. This finding was not surprising as families were more likely to support children receiving medical therapy or psychological interventions are provided. Thus, as a higher QOL is not correlated to bowel function, we assessed the children in this study at an earlier age and found that patients with comorbidities originally had low expectations, and problems with fecal incontinence may have been balanced by strong family and social support systems.

The present study had some limitations. In this single children’s medical center in South China, 398 low-type ARM cases were recorded between 2010 and 2013. However, only 21.5% of the patients with VF and 26.7% with PF were included, thereby indicating that > 70% of the affected patients could not be contacted. This may be caused by discrepancies in the postoperative follow-up system, and the charts of the patients cannot be changed to include patient contact information. However, if the patients visit surgeons regularly, their chart can be updated any time. Unfortunately, the situations may not always be ideal, and most of our patients’ parents consider poor bowel control a normal outcome, which does not require any special care. Thus, they were more likely not to visit their surgeons for follow-up after constructive surgery. A higher non-response rate may indicate that the data in this study cannot accurately convey treatment prognosis. In addition, as children grow older, their long-term bowel function and QOL must be evaluated. In future studies, the use of a matched healthy sample from a local population would be optimal for a more direct comparison between the ARM study population and healthy controls.

## Conclusions

Despite the limitations, this study is part of a new direction for middle-term bowel function and QOL evaluation of children who have undergone surgical correction for low-type ARM in China. Patients with low-type ARM can achieve good bowel control and QOL, and good postoperative anal function can lead to better QOL. Although children present with ARM with favorable outcomes, numerous children with low-type ARM still have persistent anal function problems that affect their QOL. Redo operations, mislocated anus, and incorrect treatment of constipation are the iatrogenic causes of fecal incontinence.

## Data Availability

Data sharing not applicable to this article as no datasets were generated or analysed during the current study.

## Abbreviations

ARM: anorectal malformations
BCS: Baylor Continence Scale
CG: control group
PedsQL: Pediatric Quality of Life Inventory
PF: perineal fistula
QOL: quality of life
VF: Vestibular fistula
